# Estradiol-mediated improvements in adipose tissue insulin sensitivity are related to the balance of adipose tissue estrogen receptor α and β in postmenopausal women

**DOI:** 10.1371/journal.pone.0176446

**Published:** 2017-05-04

**Authors:** Young-Min Park, Rocio I. Pereira, Christopher B. Erickson, Tracy A. Swibas, Kimberly A. Cox-York, Rachael E. Van Pelt

**Affiliations:** 1 Department of Medicine, Division of Geriatric Medicine University of Colorado Anschutz Medical Campus, Aurora, CO, United States of America; 2 Department of Medicine, Division of Endocrinology, Diabetes and Metabolism University of Colorado Anschutz Medical Campus, Aurora, CO, United States of America; 3 Denver Health and Hospital Authority, Denver, CO, United States of America; 4 Department of Nutrition, Colorado State University, Fort Collins, CO, United States of America; 5 VA Eastern Colorado Health Care System, Geriatric Research Education and Clinical Center (GRECC), Denver, CO, United States of America; Universita degli Studi di Padova, ITALY

## Abstract

We recently demonstrated that short-term estradiol (E_2_) treatment improved insulin-mediated suppression of lipolysis in postmenopausal women, but to a greater extent in those who were late compared to early postmenopausal. In this follow-up study we tested whether subcutaneous adipose tissue (SAT) expression of estrogen receptors (ER) α and β differs between early and late postmenopausal women. We further tested whether the balance of ERα to ERβ in SAT determined the effect of E_2_ on SAT insulin sensitivity. The present study included 35 women who were ≤6 years past menopause (EPM; n = 16) or ≥10 years past menopause (LPM; n = 19). Fasted SAT samples were taken following 1-week transdermal E_2_ treatment or placebo (PL) in a random cross-over design. Samples were analyzed for nuclear/cytosolic protein content and mRNA expression using Western blot and qPCR, respectively. While ESR1 increased slightly (~1.4-fold) following E_2_ treatment in both groups, ERα and ERβ protein expression did not differ between groups at baseline or in response to E_2_. However, the balance of ERα/ERβ protein in the SAT nuclear fraction increased 10% in EPM compared to a 25% decrease in LPM women (group x treatment interaction, *p*<0.05). A greater proportion of ERα/ERβ protein in the nuclear fraction of SAT at baseline (placebo day) was associated with greater reduction in SAT insulin resistance (i.e., better suppression of lipolysis, EC_50_) in response to E_2_ (r = -0.431, *p*<0.05). In conclusion, there do not appear to be differences in the proportion of adipose tissue ERα/ERβ protein in late, compared to early, postmenopausal women. However, the balance of ERα/ERβ may be important for E_2_-mediated improvement in adipose tissue insulin sensitivity.

**Trial Registration: Clinical Trials#:** NCT01605071

## Introduction

Loss of ovarian hormones and prolonged estrogen deficiency after menopause puts women at risk of metabolic dysregulation, increasing obesity and type 2 diabetes [1]. Exogenous treatment with estrogen-based hormone therapy reduces incidence of new onset diabetes in postmenopausal women [2], but whether this benefit is through improvements in insulin sensitivity remains unclear. We previously demonstrated that both conjugated estrogens and estradiol (E_2_) acutely improve insulin-mediated glucose disposal (i.e., skeletal muscle insulin sensitivity) in postmenopausal women, but only when treatment is initiated early in menopause [3–5]. Compared to early postmenopausal women, late postmenopausal women had a reduced glucose disposal after E_2_ treatment [5], supporting the concept that timing of estrogen after menopause may determine benefit versus harm for this outcome. On the other hand, insulin-mediated suppression of lipolysis (i.e., adipose tissue insulin sensitivity) was improved in both early and late postmenopausal women, suggesting benefit irrespective of time since menopause for this outcome [5]. Indeed, E_2_ improved adipose tissue insulin sensitivity to a greater extent in late compared to early postmenopausal women. The mechanism for such timing- and tissue-specific differences in E_2_ action is not known, but may be partly due to changes in estrogen receptor (ER) expression.

E_2_ exerts its biologic actions via two ER subtypes, ERα and ERβ [6]. Pre-clinical ER knockout models suggests that ERα, but not ERβ, is positively related to insulin-mediated glucose disposal and insulin signaling [7, 8]. Moreover, the overall balance of ERα relative to ERβ within a tissue is thought to be important to the action of E_2_ on insulin sensitivity [9, 10]. It is not yet known whether the balance of ERα to ERβ changes with increasing age or time since menopause and in a tissue-specific manner. We recently observed a reduced skeletal muscle ERα and ERβ nuclear protein in late, compared to early, postmenopausal women [11]. We hypothesized there would be similar reductions in adipose tissue ERα and ERβ with increasing time since menopause from these same women. We further hypothesized that the balance of ERα/ERβ in adipose tissue would be associated with insulin sensitivity and response to E_2_ in that tissue.

## Methods

### Subjects

The present study analyzed subcutaneous adipose tissue (SAT) samples from a subset of postmenopausal women who participated in a previous study [5]; samples were obtained from 35 women who were ≤6 years past menopause (EPM; n = 16) or ≥10 years past menopause (LPM; n = 19). Detailed inclusion and exclusion criteria were previously described [5]. Briefly, participants were healthy, non-obese (BMI<30kg/m^2^), sedentary to moderately active, postmenopausal women (age 45–70 yr) who had not used any estrogen-based hormone therapy. All participants provided informed consent before enrollment. This protocol was approved by the Colorado Multiple Institutional Review Board (COMIRB #11–0788).

### Study design

At the first visit, oral glucose tolerance, physical activity, and body composition were assessed. All participants underwent two treatment conditions (i.e., 1 week of transdermal estradiol [E_2_] or placebo [PL]) in a random order with a washout period (6 ± 2 wks) between study visits. On both visits, following an overnight fast, SAT was biopsied prior to insulin sensitivity assessments using hyperinsulinemic-euglycemic clamp.

### Body composition and glucose tolerance measurement

At the screening visit, fat-free mass (FFM) and total fat mass (FM) were determined by a dual x-ray absorptiometry (DXA, Hologic Discovery W, software version 11.2) as previously reported [12]. A 2hr 75g oral glucose tolerance test (OGTT) was employed in the morning following an overnight fast as previously described [5]. Circulating glucose and insulin responses over the 2hr period were measured; area under the curve (AUC) calculated using the trapezoidal method.

### Dietary lead-in period

All participants were required to maintain their body weight within ±2kg throughout testing. To control energy intake among the participants in the days prior to the testing day, a standardized diet was provided by the University of Colorado Anschutz Medical Campus (UC-AMC) Clinical Translational Research Center (CTRC) metabolic kitchen 3 days prior to each clamp study visit as previously described [5].

### Estradiol or placebo treatment

The clamp study visit was repeated following 1 week of transdermal E_2_ (0.15mg) and following matching transdermal placebo treatment in a blinded, randomized, and cross-over design.

### Hyperinsulinemic-euglycemic clamp

Under the two separate treatment conditions, participants completed a 2-stage (4 and 40 mU/m^2^/min) hyperinsulinemic-euglycemic clamp to assess insulin-stimulated glucose disposal rate (GDR) and suppression of lipolysis (EC_50_) as previously described [5]. In brief, GDR was calculated from the steady-state glucose infusion rate required to maintain euglycemia (~90mg/dL) during a 40mU/m^2^/min insulin infusion. EC_50_, or the insulin concentration to half-maximally suppress glycerol, was calculated from the exponential decay curves for glycerol across the basal and insulin stages of the clamp as previously described [13]. Plasma samples collected during the clamp visits were stored at -80°C; and analyzed for circulating glucose, insulin, and glycerol in batch by the UC-AMC CTRC laboratory.

### Human adipose tissue biopsies

On the morning prior to each clamp, SAT samples were collected from the lateral thigh region in the fasted state using a mini-liposuction technique as previously described [5]. Collected SAT was rinsed with sterile saline, separated from the fluid and immediately flash frozen in liquid N_2_ for later analyses.

### Adipocyte size and distribution

Immediately after collection, 50 mg of fresh SAT was used to determine adipocyte cell size (mean cell diameter) and size frequency distribution with Cell Counting Analysis Program (CCAP; Mayo Clinic, Rochester, MN) as previously described [14]. The relative proportion of small (20–60μ), medium (60–100μ), and large (100–140μ) adipocytes with and without treatment with 1 week of E_2_ are reported for each group.

### mRNA quantification (RT-PCR)

Total RNA was extracted from SAT samples collected using the RNeasy Lipid Mini Kit (Qiagen Inc.). RNA was 1) analyzed and quantitated using Nanodrop 2000c (ThermoFisher Scientific, Waltham, MA); 2) reverse transcribed using Verso cDNA synthesis kit (ThermoFisher Scientific, Waltham, MA), and 3) analyzed by qPCR in duplicate using TaqMan® Fast Advanced Mastermix (ThermoFisher Scientific, Waltham, MA) on an 7900HT Fast Real-Time PCR System (ThermoFisher Scientific, Waltham, MA) along with a no-template control per gene. Reference gene TATAA-box binding protein (TBP) was used as endogenous control (overall standard deviation 0.54). The Taqman primer/probes sets (Applied Biosystems, Foster, CA) used were: ESR1 (ERα, Hs00174860_m1), ESR2 (ERβ, Hs00230957_m1); and TBP Hs00427620_m1. Baseline mRNA expression was determined by calculating 2^-ΔCt^ for each sample relative to the within group mean and normalized to the reference gene. The 2^-ΔΔCt^ method was used to determine fold-changes from the placebo condition to the E_2_ condition as previously described [15].

### Cellular protein fractionation

The protocol for protein fractionation was adapted from previous research [6, 16]. Approximately 200mg AT was pulverized and homogenized in cold Buffer A containing 50mM NaF, 20mM HEPES, 100mM NaCl, 1mM ethyleneglycol tetraacetic acid (EGTA), 1mM ethylenediamine tetraacetic acid (EDTA), 1mM dithiothreitol (DTT), 2mM 4-(2-Aminoethyl) benzenesulfonyl fluoride hydrochloride (AEBSF), 1mM sodium orthovanadate, 20μg/ml aprotinin, 20μg/ml leupeptin, 20μg/ml antipain, 5mM *p*-chloromercuri phenylsulfonate, and 10mM iodoacetamide. Samples were centrifuged at 500g at 4°C for 5 min, and the supernatant (cytosolic fraction) stored at 80°C. Pellets (crude nuclei) were re-suspended and sonicated in cold Buffer B (Buffer A plus 400mM NaCl, 25% glycerol, and 0.1% sodium dodecyl sulfate [SDS]), incubated at room temperature for 15 min, then centrifuged at 3,000g at 4°C for 5 min; and the supernatant (nuclear fraction) stored at -80°C for later protein assessment.

### SDS-PAGE western blot

**Fig 1:**
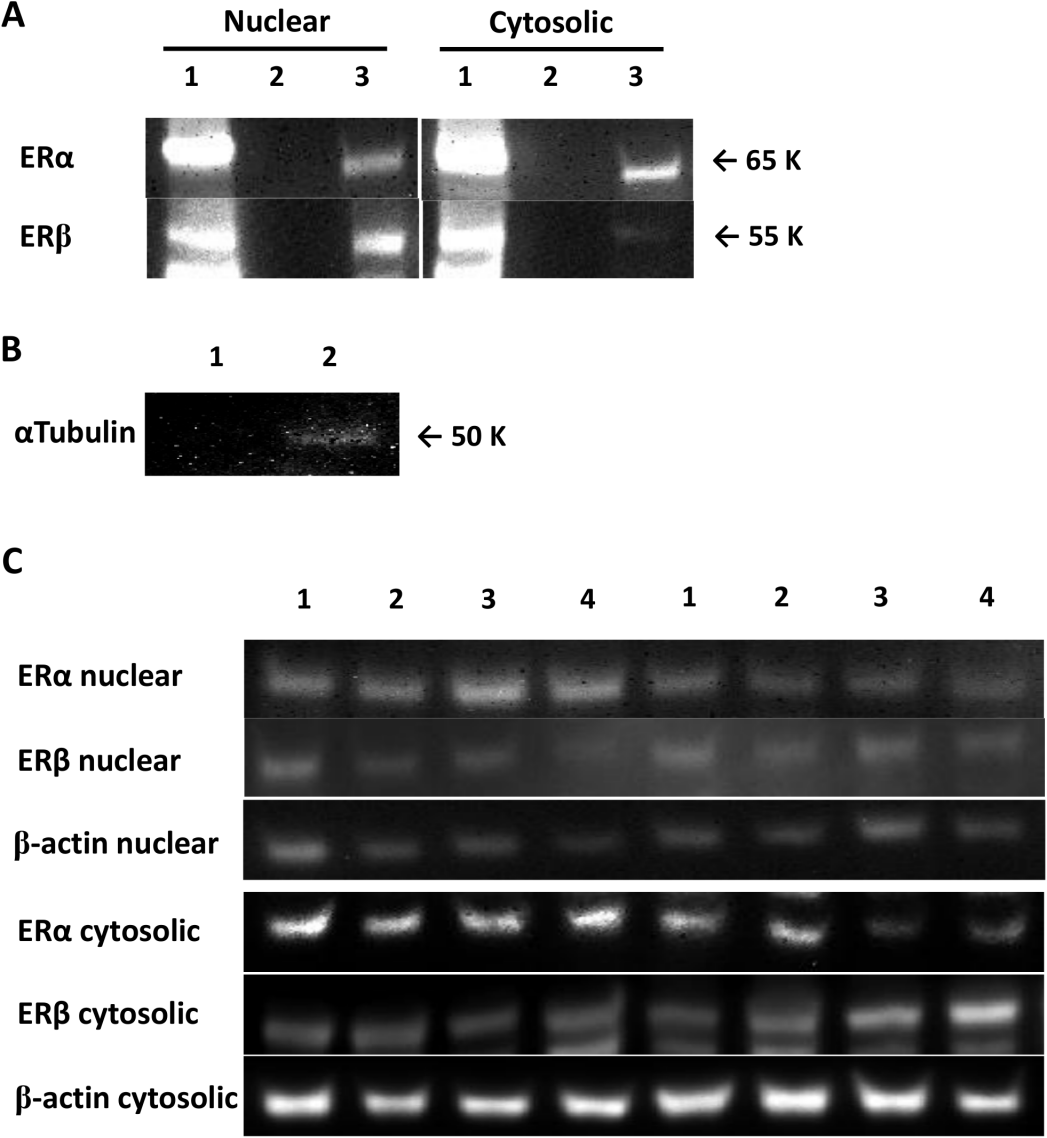
Evidence of correct western band detection for estrogen receptors (ER), the purity of nuclear fraction, and representative images. (A) ERα and β protein bands in nuclear and cytosolic fraction, Lane .1: positive control (MCF7), Lane 2: negative control (MDA-MB-231) or empty well (loading buffer only), Lane 3: 30ug protein from adipose tissue; (B) the purity of nuclear fraction, Lane 1: nuclear fraction, Lane 2: cytosolic fraction. αTubulin was used as a cytosolic marker; no bands were detected in nuclear fraction; (C) representative images of ERα and β protein bands in nuclear and cytosolic fraction.

Protein concentrations in nuclear and cytosolic fraction were measured using the Pierce BCA protein assay kit (Thermo Scientific, Rockford, IL). Samples containing the homogenates (30 μg of protein) and laemmli buffer were separated by 8% sodium dodecyl sulphate-polyacrylamide gel electrophoresis (SDS-PAGE) and transferred onto polyvinylidene difluoride (PVDF) membranes. Membranes were probed with primary antibodies (1:200–1,000 concentrations). ERα and ERβ antibodies were obtained from Cell Signaling (cs8644, Beverly, MA) and R&D systems (mab7106, Minneapolis, MN), respectively. Evidence of correct band detection for ERα and ERβ was shown in Fig 1. Individual protein bands were quantified using a densitometer (Bio-Rad Laboratories, Hercules, CA), and normalized to β-actin (loading control, #4967, Cell Signaling, Beverly, MA). Protein content was expressed relative to the early postmenopausal group placebo-treated condition. We confirmed that the results did not differ with and without this normalization step.

### Statistical analysis

The present study employed a two-group repeated measure design to test for main effects of group (EPM vs. LPM) or treatment (PL vs. E_2_) and group x treatment interactions. When a significant group x treatment interaction was found, *post hoc* tests were used for pair-wise comparisons. Pearson correlations were used to determine the associations between SAT proteins and insulin sensitivity (EC_50_). Baseline group differences (LPM vs. EPM) in descriptive outcomes were assessed by independent *t* tests. All data were analyzed using SPSS Statistics version 24.0 (IBM Corp., New York, NY). Data are reported as mean ± SEM unless otherwise specified. Level of significance was set at *P*<0.05.

## Results

### Subject characteristics

Full descriptive characteristics for women (n = 24/group) enrolled in the parent study were previously published [5]; the subset of women (n = 16-19/group) for whom we had sufficient SAT are reported herein. As reported for the full cohort, LPM women in this subset were on average 7 years older and 9 years further past menopause compared to EPM (Table 1). LPM women had less total fat and fat-free mass, but percent fat did not differ from EPM women. All women had normal fasting and post-challenge (i.e. OGTT) glucose and insulin concentrations (Table 1). Circulating E_2_, estrone, sex hormone-binding globulin (SHBG), leptin, and glycerol increased in response to E_2_ treatment (Table 2). Consistent with the parent study [5], EC_50_ was reduced in both groups (i.e., increased SAT insulin sensitivity) but to a greater extent in LPM (*P* = 0.018; Table 2).

**Table 1:** Subject characteristics.

	EPM	LPM
	n = 16	n = 19
Age (yr)	56 ± 3	63 ± 3*
Time since menopause (yr)	3 ± 1	12 ± 2*
Weight (kg)	70.5 ± 7.7	63.3 ± 8.0*
BMI (kg/m^2^)	26.1 ± 2.6	23.9 ± 3.3*
Total fat mass (kg)	26.3 ± 6.2	22.5 ± 6.3
Fat-free mass (kg)	44.3 ± 4.0	40.8 ± 3.7*
%Fat	36.9 ± 5.4	34.9 ± 6.2
OGTT glucose AUC (*10^4^)	1.2 ± 0.2	1.4 ± 0.3*
OGTT insulin AUC (*10^3^)	6.7 ± 3.9	6.3 ± 2.2

mean±SD

*p<0.05 group difference; EPM, early (≤6yr) postmenopausal; LPM, late (≥10yr) postmenopausal; BMI, body mass index; OGTT, 2hr oral glucose tolerance test; AUC, area under the curve.

**Table 2:** Treatment-associated changes in circulating hormones and metabolic markers.

	EPM		LPM		Treatment	Treatment x Group
	PL	E_2_	PL	E_2_	*p*-value	*p*-value
Baseline fasting						
E_2_ (pg/mL)	16 ± 17	179 ± 102	10 ± 6	149 ± 56	<0.0001	ns
Estrone (pg/mL)	30 ± 15	116 ± 49	29 ± 16	105 ± 48	<0.0001	ns
SHBG (nM/L)	42 ± 20	53 ± 19	57 ± 25	70 ± 31	<0.0001	ns
Leptin (ng/mL)	19 ± 13	21 ± 15	15 ± 12	18 ± 12	0.001	ns
Glucose (mg/dL)	90 ± 8	89 ± 6	89 ± 7	88 ± 10	ns	ns
Insulin (uU/mL)	8.3 ± 2.1	7.6 ± 2.6	8.7 ± 5.4	7.8 ± 3.5	0.07	ns
Glycerol (mg/dL)	121 ± 47	105 ± 32	131 ± 37	116 ± 36	<0.05	ns
FFA (umol/L)	656 ± 161	659 ± 135	793 ± 222	772 ± 182	ns	ns
During clamp (40uU/m^2^/min)						
Glucose (mg/dL)	85 ± 7	88 ± 6	88 ± 5	86 ± 6	ns	ns
Insulin (uU/mL)	91 ± 21	77 ± 16	95 ± 24	84 ± 24	0.001	ns
Glycerol (mg/dL)	34 ± 8	34 ± 13	40 ± 14	35 ± 8	ns	ns
GDR (mg/kg/min)	7.2 ± 1.8	7.3 ± 1.9	7.2 ± 2.2	6.7 ± 2.1	ns	0.10
GDR (mg/kgFFM/min)	11.5 ± 2.8	11.7 ± 3.0	11.2 ± 3.0	10.3 ± 2.8	ns	0.09
EC_50_ (uU/mL)	11.5 ± 2.1	10.6 ± 2.6	14.1 ± 5.4	11.0 ± 3.5	<0.0001	0.018

mean±SD; EPM, early (≤6yr) postmenopausal; LPM, late (≥10yr) postmenopausal; PL, placebo; E_2_, estradiol; SHBG, sex hormone-binding globulin; FFA, free fatty acid; GDR, glucose disposal rate during clamp; FFM, fat free mass; EC_50_, insulin suppression of lipolysis (insulin concentration needed to half-maximally suppress glycerol).

### Adipocyte morphology

**Fig 2:**
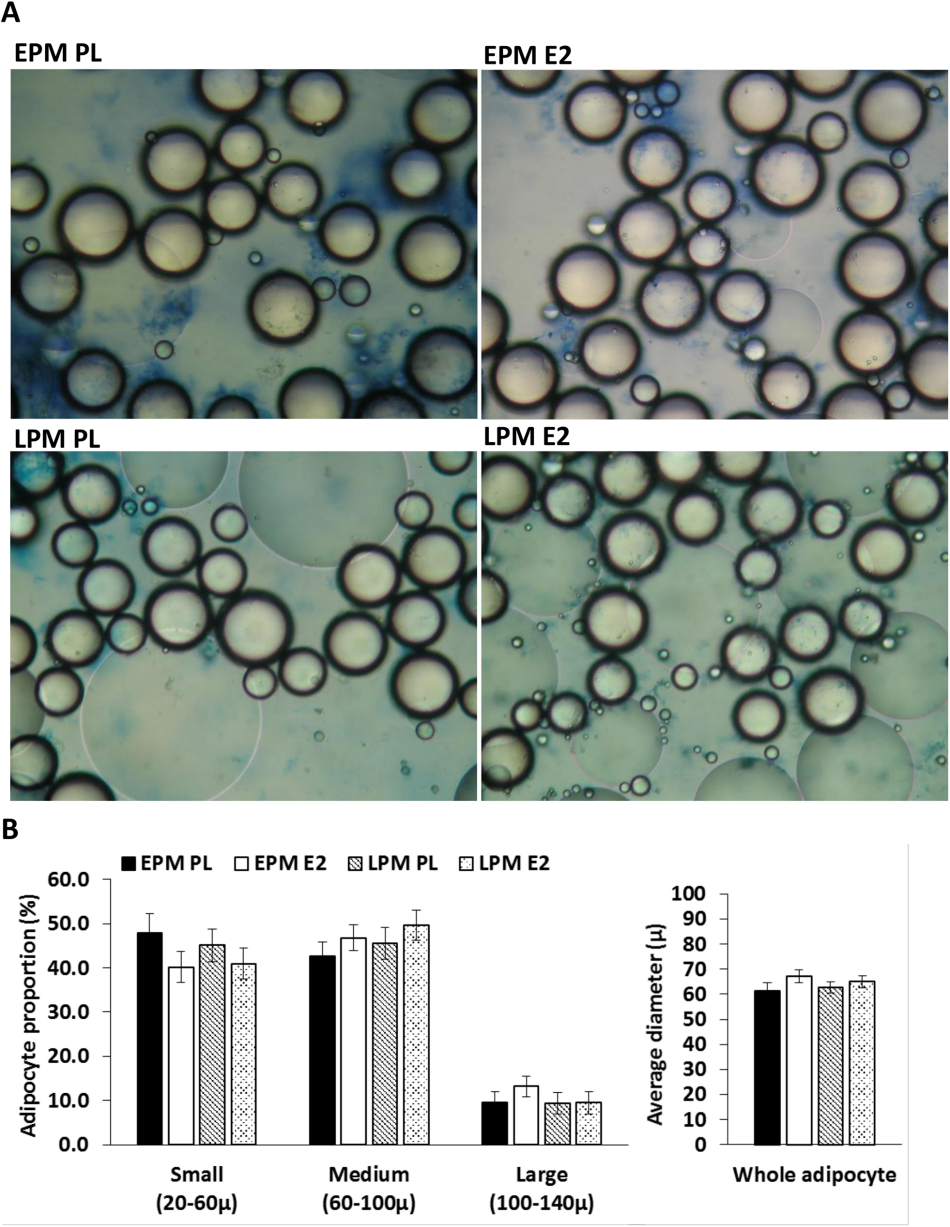
Adipocyte size and distribution. (A) Representative images of isolated adipocytes each group; (B) the proportion of small (20–60μ), medium (60–100μ), and large (100–140μ) adipocytes; and the average diameter of whole adipocytes. Values are means±SE; early postmenopausal women [EPM; n = 16]; late postmenopausal women [LPM; n = 17]; treated either with placebo [PL] or estradiol [E_2_].

The proportion of small, medium, and large adipocytes over whole adipocytes, and the average diameter of whole adipocytes were not statistically different between groups. However, there was a non-significant trend for the proportion of small adipocytes to be decreased (*P* = 0.11; Fig 2B) and the average diameter of adipocytes to be increased (*P* = 0.10; Fig 2B) in response to E_2_ treatment across groups.

### Baseline (placebo) group difference

**Fig 3:**
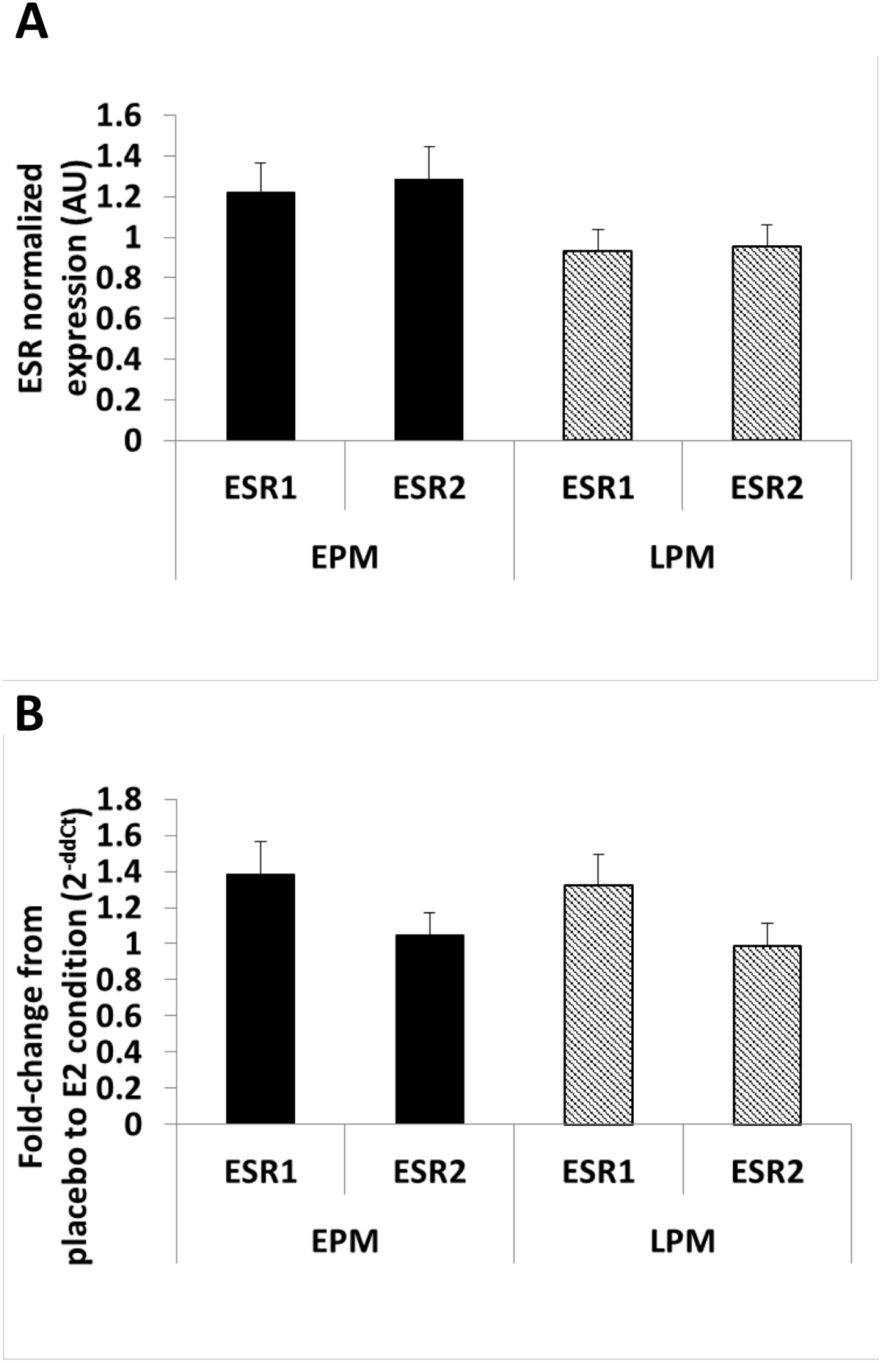
Estrogen receptor (ER) gene expression. (A) Baseline (placebo) group difference for ESR1 (ERα) and ESR2 (ERβ) gene expression; (B) fold-change from placebo condition in response to 1 week of transdermal E_2_ treatment. Values are means±SE; early postmenopausal women [EPM; n = 12]; late postmenopausal women [LPM; n = 16].

**Fig 4:**
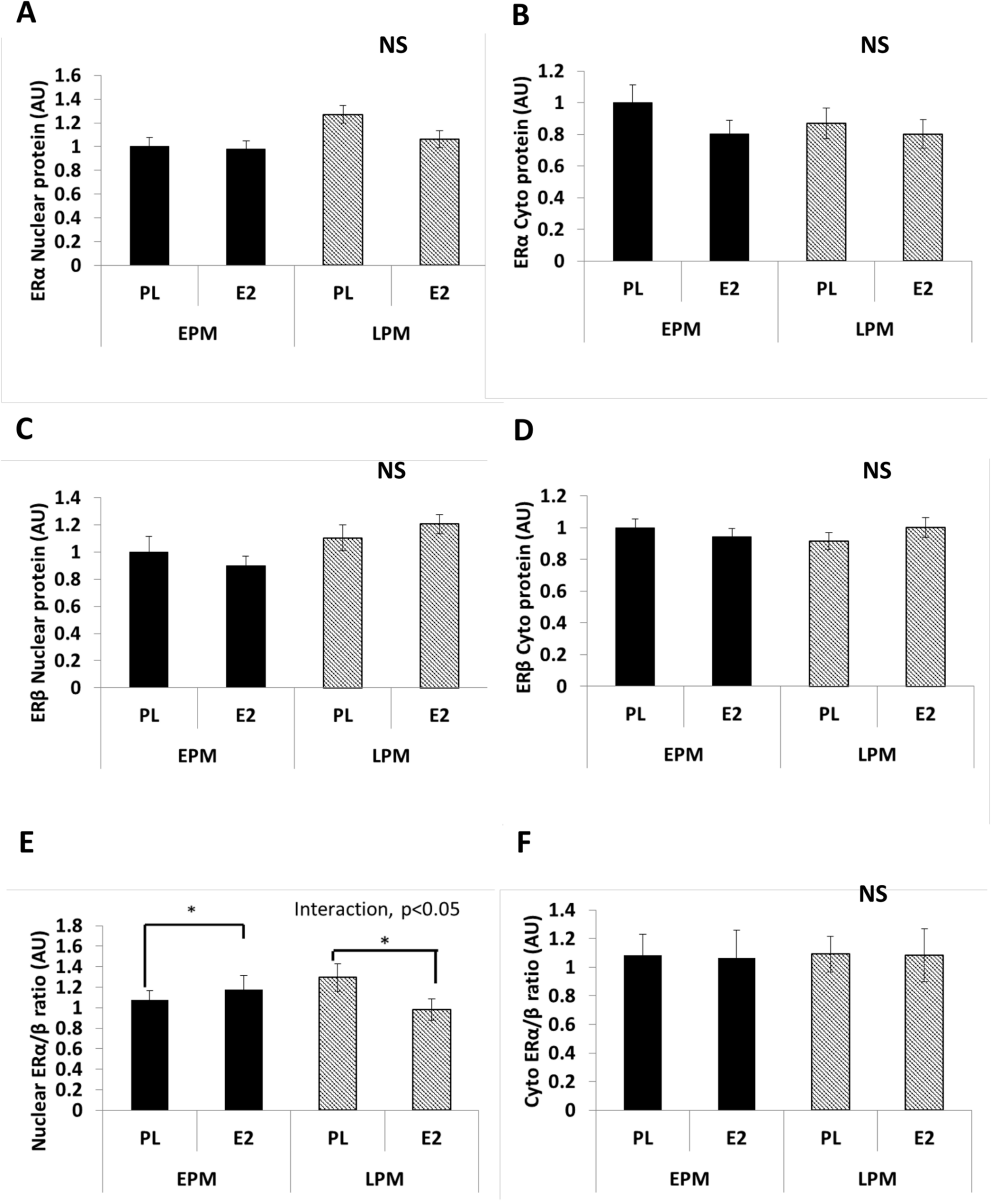
Estrogen receptor (ER) protein content. (A) ERα protein in nuclear fraction, (B) ERα protein in cytosolic fraction, (C) ERβ protein in nuclear fraction, (D) ERβ protein in cytosolic fraction, (E) ratio of ERα/ERβ protein in nuclear fraction, and (F) ratio of ERα/ERβ protein in cytosolic fraction. Values are means±SE (n = 14–19 per group). NS = no significant difference between groups; Interaction = group-by-treatment interaction; group (early postmenopausal women [EPM] vs. late postmenopausal women [LPM]); treatment (estradiol [E_2_] vs. placebo [PL]). *Denotes significant (*P*<0.05) within group change in response to E_2_ treatment

There were no group (LPM vs. EPM) differences in SAT ESR1 (ERα; *P* = 0.102) or ESR2 (ERβ; *P* = 0.078; Fig 3A) gene expression. There were also no group differences in the amount of ERα (Fig 4A and 4B), ERβ (Fig 4C and 4D), or the proportion of ERα/ERβ (Fig 4E and 4F) protein in either the nuclear or cytosolic fractions of SAT.

### Changes in response to E_2_

Adipose tissue ESR1, but not ESR2, gene expression increased 1.4- and 1.3- fold in response to E_2_ treatment in EPM and LPM, respectively (Fig 3B). ERα and ERβ protein content in the nuclear and cytosolic fractions did not change in response to E_2_ treatment, but the proportion of ERα/ERβ in the nuclear fraction was increased in EPM and decreased in LPM (Fig 4; interaction, *P* = 0.04). There was no change in cytosolic ERα/ERβ following E_2_ treatment.

### Correlations

**Fig 5:**
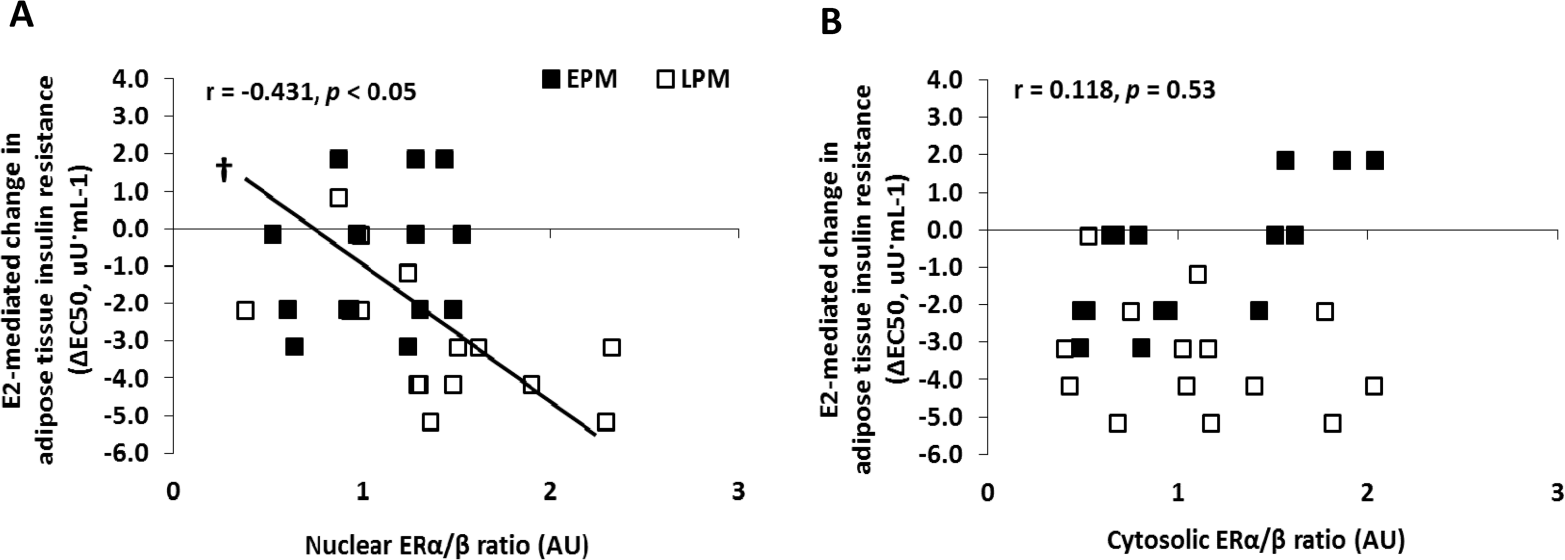
Estradiol (E_2_)-mediated change in adipose tissue insulin resistance versus the ratio of estrogen receptor (ER) α/β protein. Association between E_2_-mediated change in adipose tissue insulin resistance (ΔEC_50_) and baseline (placebo): (A) nuclear ERα/ERβ; or (B) cytosolic ERα/ERβ. **†**Denotes a significant (*P*<0.05) correlation.

The E_2_-mediated change in SAT insulin resistance was inversely correlated with the proportion of ERα/ERβ protein in the nuclear fraction (r = -0.431, *P* = 0.017; Fig 5A), but not the cytosolic fraction (r = 0.118, *P* = 0.535; Fig 5B) at baseline (placebo day).

## Discussion

The present study is the first to show that adipose tissue ERα and ERβ protein are not reduced in late, compared to early, postmenopausal women. However, those women who had a greater proportion of ERα/ERβ in adipose tissue showed a greater improvement in adipose tissue insulin sensitivity (i.e., less insulin needed to suppress lipolysis) in response to E_2_. While ERα and ERβ protein did not change in response to 1 week of E_2_, the proportion of ERα/ERβ increased 10% in early postmenopausal women compared to a 25% decrease in late postmenopausal women.

### Differences between adipose tissue and muscle

The results of the current studies in adipose tissue differ from our recent studies in skeletal muscle. In muscle we observed a reduction in both ERα and ERβ protein in late, compared to early, postmenopausal women suggesting declines with time since menopause [11]. Conversely, in the present study the lack of a group difference in adipose tissue ERα and ERβ suggested there may not be an inevitable decline in adipose tissue ER with increasing time since menopause. Similar to what we previously observed in muscle, 1 week of E_2_ did not change ER protein expression in adipose tissue. However, unlike muscle, the balance of ERα/ERβ in adipose tissue did appear to change in response to E_2_. This might suggest that longer term use of estrogens could eventually impact overall expression of ER in adipose tissue. Another difference between adipose tissue and skeletal muscle that emerged was in the association between ER expression and insulin sensitivity within that tissue. In the current study the balance of ERα/ERβ in adipose tissue predicted E_2_-mediated changes in adipose tissue insulin sensitivity (i.e., insulin suppression of lipolysis). Whereas in skeletal muscle the prevailing balance of ERα/ERβ was not predictive of E_2_-mediated changes in skeletal muscle insulin sensitivity (i.e., insulin stimulated glucose uptake).

### Balance of estrogen receptors and tissue function

E_2_ exerts biologic actions through two receptor sub-types, ERα and ERβ [6]; the metabolic action of which is often opposing. Global knockout of ERα, but not ERβ, leads to a metabolic syndrome phenotype (e.g., impaired glucose homeostasis, inflammation) in rodent models [10]. Global knockout models further suggest that E_2_ binding to ERα protects against increasing adiposity, while E_2_-activated ERβ appears to accelerate adiposity [17]. Moreover, knocking out ERα in adipose tissue specifically leads to adipose tissue dysfunction (i.e., inflammation and fibrosis); and in the absence of ERα, ERβ appears to play a protective role [18]. Thus the balance of the two ER subtypes is thought to be important to adipose tissue function and metabolic phenotype.

### Changes in estrogen receptor balance over time

Few studies have measured ER in human tissues, so it is not yet known whether the balance of ERα/ERβ changes with age, as women transition through menopause, or with prolonged estrogen deficiency after menopause. One cross-sectional study reported that ERβ, but not ERα, was higher in postmenopausal compared to premenopausal women [19]. In the present study we found no differences in adipose tissue ERα and ERβ in late compared to early postmenopausal women. In the context of our previously observed declines in skeletal muscle ERα and ERβ with increasing time since menopause, it appears that any changes in ER with aging or estrogen deficiency are likely tissue-specific. Future studies are greatly needed to examine whether the relative balance of ERα/ERβ changes with aging or estrogen deficiency in other E_2_-sensitive tissues and cells (e.g., brain, liver, immune cells).

### Estrogen receptor response to exogenous E_2_

In the present study we observed an increase in *ESR1* gene expression following one week of transdermal E_2_ in both early and late postmenopausal women; yet ERα protein did not change in response to E_2_. This discrepancy between gene expression and protein may be due to the posttranslational modification (e.g., protein degradation or ubiquitination) [20], resulting in no protein difference between groups shown in the present study. Despite lack of significant change in either ERα or ERβ separately, there was a group-by-treatment interaction such that the balance of nuclear ERα/ERβ protein increased in early but decreased in late postmenopausal women. If such acute changes in ERα/ERβ persist with longer term estrogen treatment, this might prove beneficial to early postmenopausal women but harmful to late postmenopausal women. Future studies are needed to determine how long term use of estrogen-based hormone therapies impacts the balance of ER across tissues and with increasing time since menopause.

### Adipose tissue estrogen receptors and insulin action

The importance of ERα for glucose homeostasis and skeletal muscle insulin sensitivity has been well documented in pre-clinical studies [9, 10]. By comparison little is known about the relative importance of ER for adipose tissue insulin sensitivity. Knocking out ERα promotes increased adiposity, possibly secondary to adipose tissue dysfunction [10, 18]. Changes in adipose tissue in response to ERα knockout include loss of GLUT4 (glucose transporter protein) and increased inflammation and fibrosis [18, 21]. However, to our knowledge, adipose tissue sensitivity to insulin suppression of lipolysis has not been studied in these preclinical models. In premenopausal human tissue there is evidence to suggest that E_2_, via ERα, may impact adipose tissue lipolysis via increased expression of adrenergic receptors (i.e., increasing sensitivity to adrenergic stimulation) [22]. Our studies in postmenopausal human tissue further suggest that E_2_ impacts adipose tissue lipolysis via increased EC_50_ for glycerol (i.e., increasing sensitivity to insulin stimulation). Future studies are needed to determine whether such E_2_-mediated improvements in lipolytic sensitivity play a role in adipose tissue accrual and function. Moreover, given the relative importance of well-functioning adipose tissue on metabolic health [23], it would be useful to know whether E_2_-mediated improvements on whole body glucoregulatory insulin sensitivity are secondary to improvements in adipose tissue lipolytic sensitivity.

### Potential limitations

There are some limitations in the current study that should be considered. First, we used the EC_50_, or insulin concentration to half-maximally suppress lipolysis (glycerol), as an index of adipose tissue insulin sensitivity. The measures of insulin and glycerol are done at the whole body level rather than at the tissue level, so the source of glycerol is not exclusively from adipose tissue. However, the contribution of glycerol from muscle and liver is negligible [24] and the systemic measurement of lipolysis is preferable to in vitro where it is difficult to precisely replicate the in vivo insulin and estrogen conditions. Second, in the present study we only collected adipose tissue form the femoral depot, so we did not have abdominal adipose tissue for comparison. Our previous studies suggest there are not depot (abdominal vs. femoral) differences in adipose tissue sensitivity to insulin suppression of lipolysis in postmenopausal women [25]. Nevertheless, there may have been depot differences in ER expression that may have impacted our results. Third, this is a follow-up study to analyze tissues obtained from a larger trial which was powered to detect group differences in E_2_-mediated alterations in whole body glucose disposal; thus the variables reported herein were not statistically powered *a priori*. Fourth, we detected Lamin (nuclear protein marker; S1 Fig) in the cytosolic fraction from a number of our SAT samples, suggesting our cytosolic fractions had nuclear contamination possibly due to the freezing process. However, we did not find α-Tubulin (cytosolic protein marker; Fig 1) in the nuclear fraction so these samples were pure, giving us confidence in our nuclear ER protein results.

### Conclusions

Our data suggest that there is not an inevitable decline in adipose tissue ERα and ERβ protein with increasing age or duration of estrogen deficiency. Compared to placebo, one week of transdermal E_2_ improved adipose tissue insulin sensitivity (i.e., less insulin needed to suppress lipolysis) in postmenopausal women, particularly in those women who had a greater proportion of ERα/ERβ in adipose tissue. Additional well-controlled human studies are needed to determine the mechanism by which the balance of ERα/ERβ impacts the physiologic action of E_2_ in all estrogen-responsive tissues.

## Supporting information

S1 Fig Nuclear protein marker in nuclear and cytosolic fraction. (PPTX)
