# Supplementary figures and images for: Estradiol-mediated improvements in adipose tissue insulin sensitivity are related to the balance of adipose tissue estrogen receptor α and β in postmenopausal women
Source: PLoS One. 2017 May 4;12(5):e0176446. doi: 10.1371/journal.pone.0176446 (PMC5417515; doi:10.1371/journal.pone.0176446)

## Slide 1
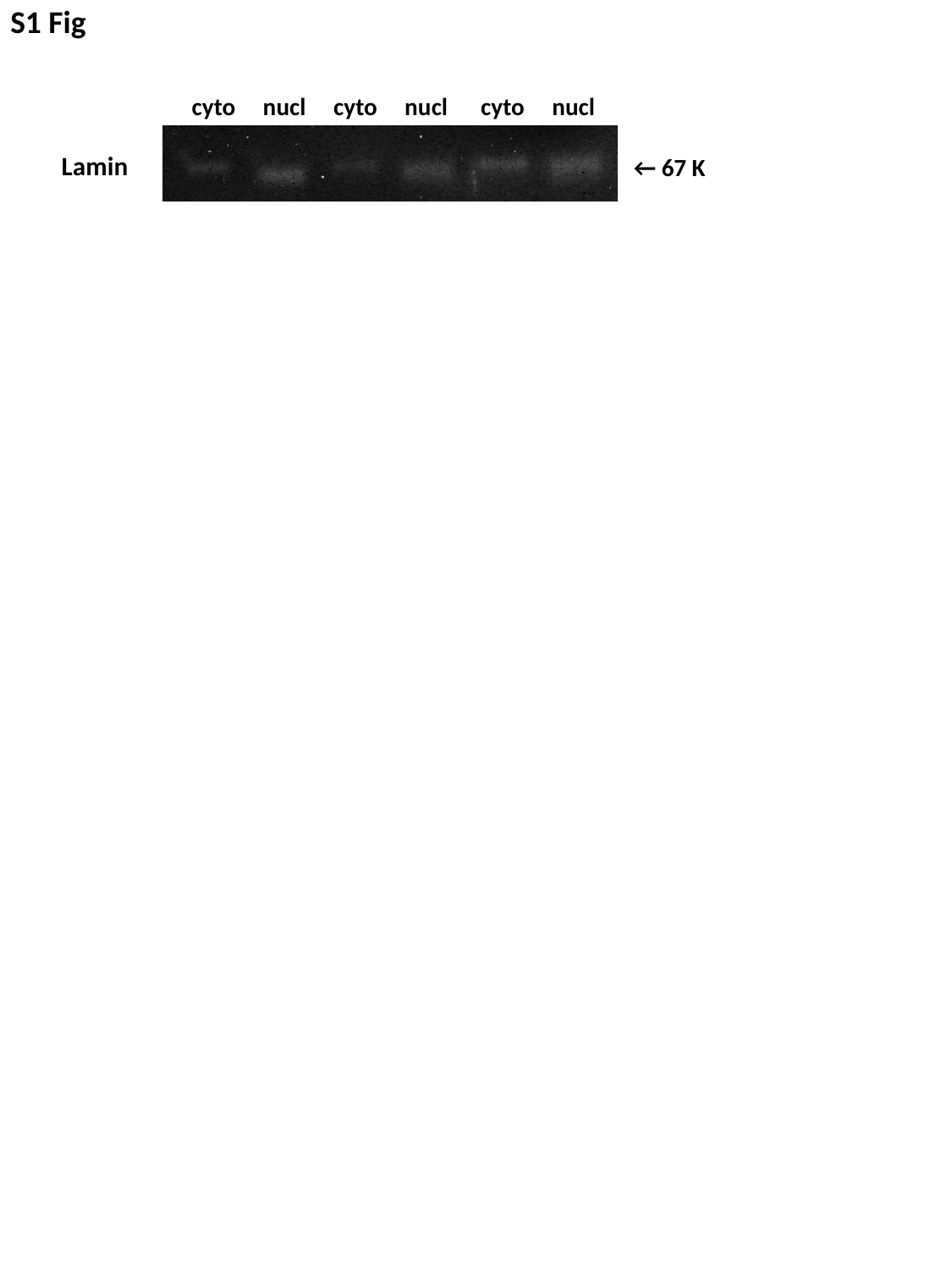

S1 Fig
 cyto nucl cyto nucl cyto nucl
Lamin
← 67 K

Supplement: S1 Fig — (PPTX) [file pone.0176446.s001.pptx]
